# MicroRNA-506-3p targets SIRT1 and suppresses AMPK pathway activation to promote hepatic steatosis

**DOI:** 10.3892/etm.2022.11390

**Published:** 2022-05-23

**Authors:** Liang-Kai Hu, Jian-Qing Chen, Hao Zheng, Yuan-Ping Tao, Yuan Yang, Xuan-Fu Xu

Exp Ther Med 22:1430, 2021; DOI: 10.3892/etm.2021.10865

Following the publication of this article, the authors regret that the paper contained some errors that should have been corrected before the article went to press. First, in the Materials and methods section, “*miRNA transfection*” subsection, on p. 2, right-hand column, line 6, the text “miR-505-3p mimics (5’-GGGAGCCAGGAACGAUUGAUGU-3’), inhib-itor (5’-ACUACUGAGCCGCAGUAGA-3’)” should have been written as “miR-506-3p mimics (5’-UAAGGCACCC-UUCUGAGUAGA-3’), inhibitor (5’-UCUACUCAGA-AGGGUGCCUUA-3’)”. Secondly, the authors have subsequently realized that certain of the primer sequences featured in Table I were written erroneously; specifically, the sequences for SREBP1, FASN, SCD1 and ACC1. A corrected version of Table I is shown opposite, where the proper sequences for SREBP1, FASN, SCD1 and ACC1 are shown, highlighted in bold.

The authors are grateful to the Editor of *Experimental and Therapeutic Medicine* for allowing them the opportunity to publish this corrigendum, and allt he authors agree with its publication. The authors also regret any inconvenience that this mistake has caused.

## Figures and Tables

**Table I:** Sequences of primers used for reverse transcription-quantitative PCR.

Primer	Sequence (5'-3')
SIRT1	F: TGCGGGAATCCAAAGGATAA
	R: CAGGCAAGATGCTGTTGCA
miR-506-3p	F: TAAGGCACCCTTCTGAGTAGA
	R: GCGAGCACAGAATTAATACGAC
SREBP1	F: **CCAGGGCAGGACACGAAC**
	R: **TGAAGGGTGGCTCGTCCAT**
FASN	F: **ATGAGCACCAACGACACGAT**
	R: **GGTTCAGGAAGAGGTCCAGC**
SCD1	F: **GAAGACGACATTCGCCCTGA**
	R: **CCATACAGGGCTCCCAAGTG**
ACC1	F: **AACGGAGGCTGGGAAAATGG**
	R: **TGGAGTGTCCTTTCTGGTCAAC**
U6	F: CTCGCTTCGGCAGCACA
	R: AACGCTTCACGAATTTGCGT
GAPDH	F: TGTGGGCATCAATGGATTTGG
	R: ACACCATGTATTCCGGGTCAAT

SIRT1, sirtuin 1; miR, microRNA; SREBP1, sterol regulatory element-binding protein 1; FASN, fatty acid synthase; SCD1, stearoyl-CoA desaturase-1; ACC1, acetyl-CoA carboxylase 1; F, forward; R, reverse.

